# Symptoms and yield loss caused by rice stripe mosaic virus

**DOI:** 10.1186/s12985-019-1240-7

**Published:** 2019-11-27

**Authors:** Siping Chen, Weilin Li, Xiuqin Huang, Biao Chen, Tong Zhang, Guohui Zhou

**Affiliations:** Key Laboratory of Microbial Signals and Disease Control of Guangdong Province, College of Agriculture, South China Agricultural University, Guangzhou, 510642 Guangdong China

**Keywords:** Rice viral disease, Rice stripe mosaic virus, Symptomology, Yield loss assessment

## Abstract

**Background:**

Rice stripe mosaic virus (RSMV) is a tentative new *Cytorhabdovirus* species in family *Rhabdoviridae* transmitted by the leafhopper *Recilia dorsalis*. Although the virus was first detected in southern China in 2015, few studies have investigated rice symptoms and yield losses caused by RSMV infection.

**Methods:**

In this study, we observed and systematically compared symptoms of three virally infected, representative varieties of *indica*, hybrid and *japonica* rice and determined the yield parameters of the artificially inoculated plants.

**Results:**

The three RSMV-infected cultivated rice varieties exhibited slight dwarfing, striped mosaicism, stiff, crinkled or even twisted leaves, an increased number of tillers, delayed heading, cluster-shaped shortening of panicles and mostly unfilled grains. Slight differences in symptom occurrence time were observed under different environmental conditions. For example, mosaic symptoms appeared earlier and crinkling symptoms appeared later, with both symptoms later receding in some infected plants. Yield losses due to RSMV also differed among varieties. The most serious yield reduction was experienced by *indica* rice (cv. Meixiangzhan), followed by hybrid *indica* rice (cv. Wuyou 1179) and then *japonica* (cv. Nipponbare). Single panicle weight, seed setting rate and 1000-kernel weight were reduced in the three infected varieties compared with healthy plants—by 85.42, 94.85 and 31.56% in Meixiangzhan; 52.43, 53.06 and 25.65% in Wuyou 1179 and 25.53, 49.32 and 23.86% in Nipponbare, respectively.

**Conclusions:**

Our findings contribute basic data for field investigations, formulation of prevention and control strategies and further study of the pathogenesis of RSMV.

## Background

Rice (*Oryza sativa*) is one of the world’s major cereal food crops. Rice production is seriously threatened by viral diseases [1], which have become an important problem. As many as 17 rice viruses, widely distributed in rice production areas of Asia and Africa, have been reported. Most of these viruses are transmitted by insects and cause intermittent outbreaks [2–4].

Rice stripe mosaic virus (RSMV) is a tentative new species first detected in southern China in 2015. RMSV is the first reported *Cytorhabdovirus* (family *Rhabdoviridae*) transmitted by the leafhopper *Recilia dorsalis* and the first to infect rice. Previous research has shown that the virus is widely distributed in southern China, where the incidence of infection in some fields exceeds 70% and causes serious rice production losses [2].

RSMV-infected rice is mainly characterized by slight dwarfing, the presence of twisted leaves exhibiting striped mosaicism, an increased number of tillers, inferior heading and mostly unfilled grains [2]. Although these symptoms have been reported, previous studies of RSMV have not addressed the viral disease symptom process, symptomatic differences among rice varieties or effects on yield components. In this study, we accordingly investigated RSMV symptoms and their effects on yield factors of representative *indica*, hybrid and *japonica* rice varieties (cultivars Meixiangzhan, Wuyou 1179 and Nipponbare, respectively) following artificial inoculation in an insect-proof room. Our findings can serve as a basis for disease diagnosis in the field and the formulation of control strategies.

## Materials and methods

### Rice, virus and vector insect

Three representative rice varieties, *indica* rice Meixiangzhan, *japonica* rice Nipponbare and hybrid *indica* rice Wuyou 1179, were purchased from a commercial supplier (Guangdong South China Agricultural University Seeds, China). Plants infected with RSMV and *R. dorsalis* leafhoppers were collected in Luoding, Guangdong Province, China, and maintained in a greenhouse in our laboratory [5].

### Rice culture

Seeds of the tested rice varieties were soaked in water for 12 h, germinated for 2 days at 37 °C, and then sown in organic peat soil (Jiffy). The germinated seedlings were grown in a greenhouse at 28 °C and 60% relative humidity under long-day conditions (16-h light/8-h dark) until the three-leaf stage [6]. Seedlings at the same growth stage were selected for inoculation with RSMV.

### Virus inoculation and detection

Caged, late-stage nymphs of *R. dorsalis* were allowed to feed on RSMV-infected rice plants at the tillering stage for 16 days. Test seedlings of rice at the three-leaf stage were then inoculated using viruliferous (RSMV) or virus-free (mock) leafhoppers at a ratio of three insects per plant for 3 days. After removal of insects, the inoculated plants were transplanted into a round area with a diameter of 29 cm per plant in an insect-proof room and cultured to maturity at 25 °C − 30 °C and 60–80% relative humidity under long-day conditions (16-h light/8-h dark). The experiment was carried out from April to May 2018, with three biological replicates processed independently. At least 30 plants per rice variety were included in each replicate.

Total RNA was extracted from new leaf tissue (~ 100 mg) taken from the upper part of rice seedlings at 15 dpi using Trizol reagent according to the manufacturer’s instructions (Vazyme Biotech, China). Reverse transcription polymerase chain reaction (RT-PCR) amplification of the viral RNA was carried out using a HiScript II One-Step RNA PCR kit (Dye Plus) P612–01(Vazyme Biotech), and the virus was detected using a procedure similar to a previously reported method [2]. Virus-positive plants were defined as infected, while virus-negative plants were considered to be healthy. The plants were monitored for symptoms, and yield factors were measured.

### Observation of symptoms and measurement of relevant indicators

Symptoms of tested rice plants were observed, plant heights measured and symptomatic rates counted at 5, 10, 15, 20, 25, 30, 45 and 60 dpi. When 100% of healthy control plants had headed, the number of heading virus-infected plants was measured and the heading rate of the infected plants was calculated.

A SPAD-502 Plus chlorophyll meter (Minolta, Osaka, Japan) was used to determine the chlorophyll content of upper, middle and lower portions of tested rice plant leaves at 45 dpi. The average SPAD value of the three leaf parts was used as an indicator of leaf chlorophyll content [7].

At 45dpi, the middle part of the test plant leaves (~ 5 g) were collected and dried at 65 °C for 48 h. After pulverization, the samples were passed through 40- to 80-mesh sieves and extracted with a 2:1 mixture of acetone and alcohol. After carbonization of cellulose with 72% sulfuric acid, the extract was diluted with distilled water and heated at 121 °C for 1 h. The hydrolysate was filtered, and cellulose, hemicellulose and lignin contents were measured using a 940 Professional IC Vario instrument. Further, the data was converted cellulose and hemicellulose content according to National Renewable Energy laboratory’s standard [8].

### Yield loss determination

At rice plant maturity, the following parameters were measured: the number of tillers, plant height, panicle length, effective panicle number, filled grain number, empty grain number, total grain number, single panicle weight and kernel weight.

### Data analysis

The statistical software package SPSS 20.0 (IBM, Armonk, NY, USA) was used to analyze the experimental data. The data were analyzed by one-way analysis of variance (ANOVA) to check for significant differences between infected and healthy plants at *P* < 0.05 and *P* < 0.01. Pairwise differences between different cultivars and the significant differences between infected and healthy plants in percentage were evaluated using the nonparametric Kruskal-Walli tests.

## Results

### Characteristic symptoms

In our three experiments, the RSMV-infected Meixiangzhan plants that we obtained were respectively 25(83.33%), 26(86.67%) and 18(60%); and RSMV-infected Nipponbare plants were respectively 28(93.33%), 26(86.67%) and 26(86.67%); and RSMV-infected Wuyou 1179 plants were respectively 28(93.33%), 27(90.00%) and 22(73.33%). The infected plants were used for the observation of symptoms and measurement of relevant indicators.

**Fig. 1:**
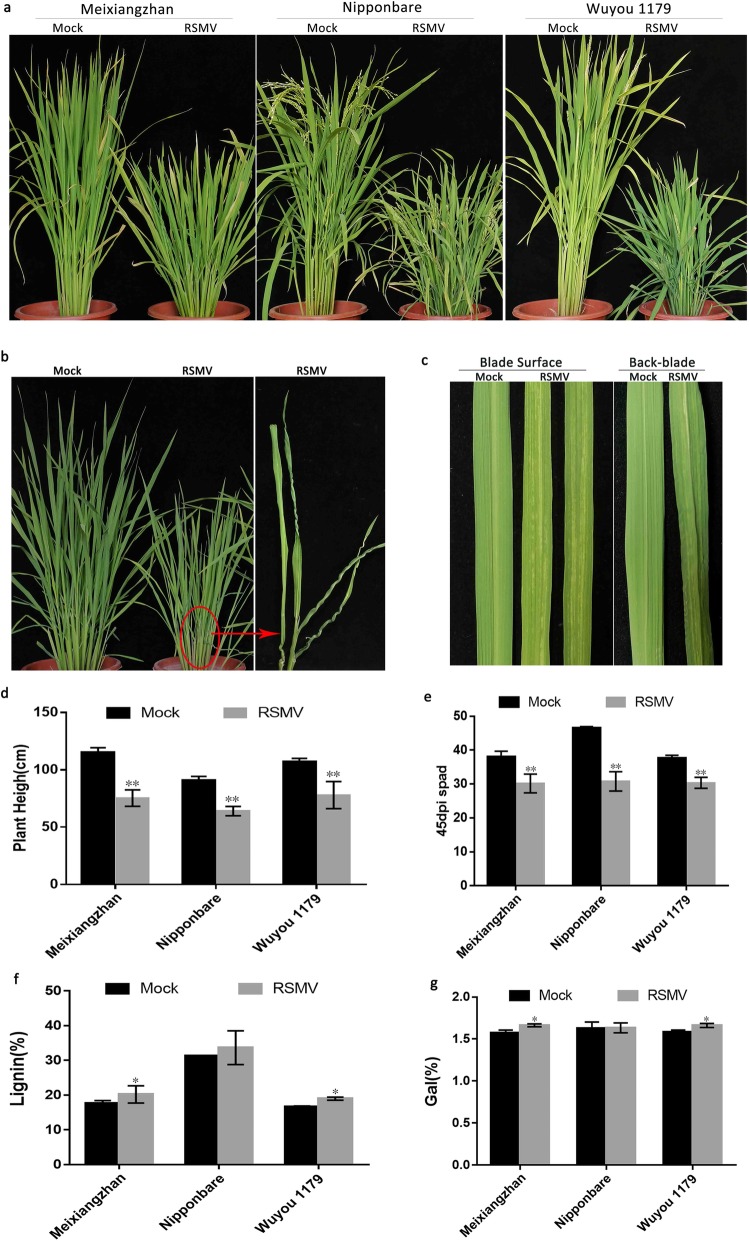
Typical symptoms of RSMV infection in rice. **a** Plant heights of three RSMV-infected varieties at 75 dpi. **b** Stiff, twisted leaves of RSMV-infected Wuyou 1179 at 45 dpi. **c** Mosaicism on leaf blade surfaces and undersides of Meixiangzhan at 45 dpi. **d** Plant heights of the three RSMV-infected varieties at 60 dpi. (Meixiangzhan: df = 22.035, SD = 1.98, *p* < 0.0001; Nipponbare: df = 28, SD = 1.34, *p* < 0.0001; Wuyou 1179: df = 15.705, SD = 2.91, *p* < 0.0001) **e** SPAD values of the three infected varieties at 45 dpi. (Meixiangzhan: df = 26.036, SD = 1.057, *p* < 0.0001; Nipponbare: df = 17.860, SD = 1.34, *p* < 0.0001; Wuyou 1179: df = 28, SD = 0.86, *p* < 0.0001) **f–g** Leaf lignin (**f**) and galactose (**g**) contents of the three infected varieties (Leaf lignin Contents: Meixiangzhan: df = 1, *p* = 0.05; Nipponbare: df = 1, *p* = 0.513; Wuyou 1179: df = 1, *p* = 0.05; Leaf galactose Contents: Meixiangzhan: df = 1, *p* = 0.05; Nipponbare: df = 1, *p* = 0.827; Wuyou 1179: df = 1, *p* = 0.05) Values in d–g are means ± s.d. (d–e, *n* = 15 plants; f-g, *n* = 3 replicates). Significant differences are indicated by asterisks: *, significant (*P* < 0.05); **, highly significant (*P* < 0.01)

Typical RSMV symptoms of plant dwarfing and striped or spotted, stiff, crinkled or even twisted leaves were observed in the three tested varieties (Fig. 1a–c). No significant difference in the types of symptoms was noted among varieties, but the severity of symptoms varied.

Plant heights of rice cultivars Meixiangzhan, Nipponbare and Wuyou 1179 at 75 days post infection (dpi) with RSMV were respectively 65.41, 70.75 and 72.84% of those of healthy control plants, and the infected plants displayed significant dwarfing (Fig. 1d). The three rice varieties were dwarfed to different extents after RSMV infection: Meixiangzhan exhibited the highest degree of dwarfing, followed by Nipponbare and then Wuyou 1179 (Additional file 1: Figure S1).

Leaf chlorophyll contents, as reflected by SPAD values, of the infected varieties Meixiangzhan, Nipponbare and Wuyou 1179 were reduced respectively by 20.71, 33.86 and 19.40% of those of healthy control plants at 45 dpi, thus indicating that the chlorophyll contents of infected plants were obviously decreased (Fig. 1e). The degree of reduction differed significantly among the three varieties; the highest reduction in SPAD values was in Nipponbare, followed by Meixiangzhan and then Wuyou 1179 (Additional file 2: Figure S2).

Cellulose, hemicellulose and lignin contents of plant leaves affect leaf morphology. Infection with RSMV can increase the stiffness of rice leaves, a change that is readily apparent by manual comparison of infected and healthy leaves. At 45 dpi, lignin contents of the three tested varieties were increased (Fig. 1f). Cellulose contents of Meixiangzhan and Wuyou 1179 were decreased, and the hemicellulose contents of three varieties were also decreased (Additional file 15: Table S1). Glucose contents of *indica* and hybrid rice varieties decreased by 9.32 and 6.87%, respectively (Additional file 3: Figure S3). Galactose contents of the three tested varieties (Meixiangzhan, Nipponbare and Wuyou 1179) increased by 5.69, 0.24 and 5.01%, respectively (Fig. 1g). Changes in the contents of these substances may be responsible for the stiffening of leaves.

### The process of characteristic symptom development

**Fig. 2:**
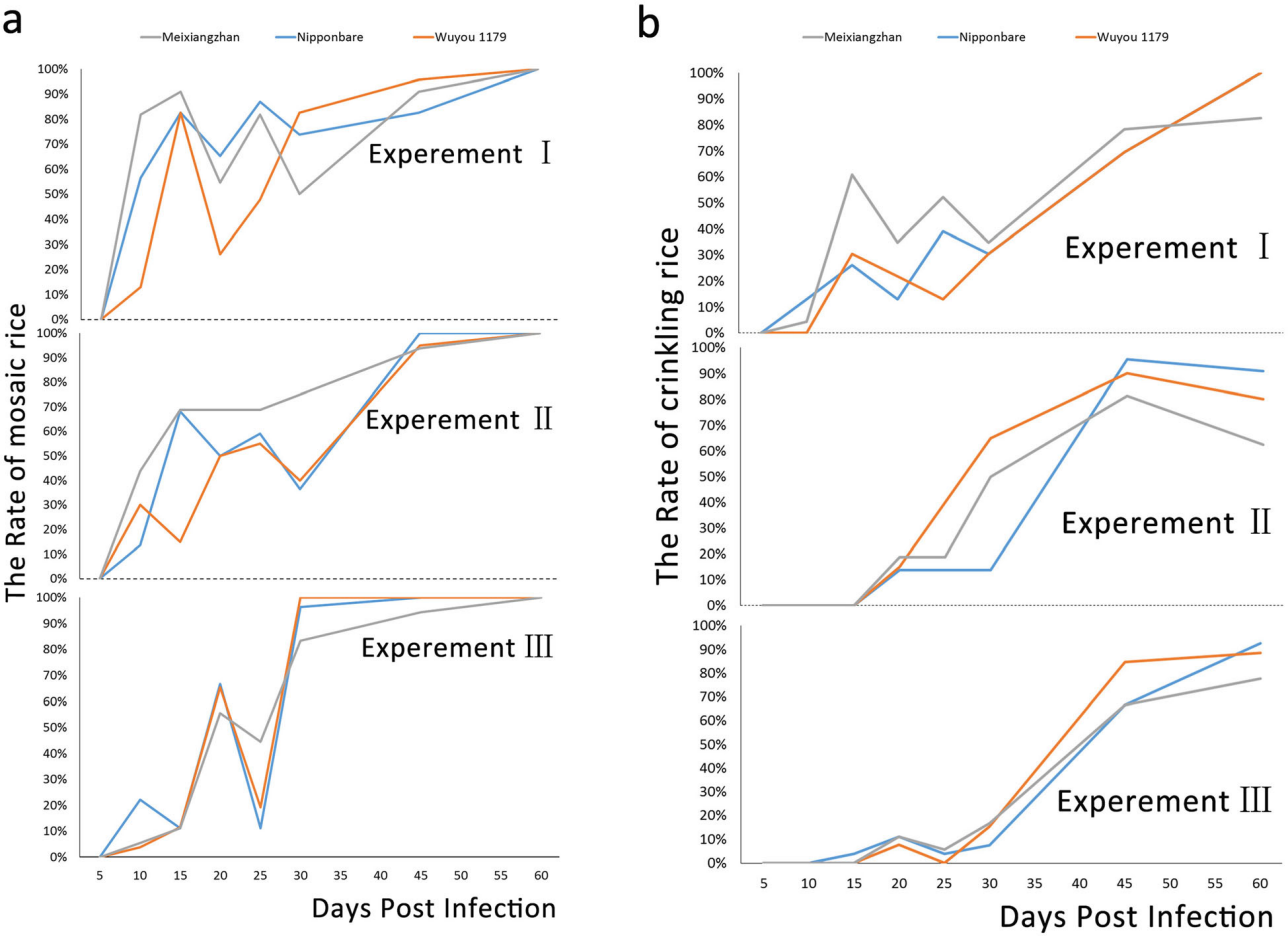
Development of characteristic symptoms in three rice varieties after infection with RSMV. a–b Proportion of plants with mosaic (**a**) and leaf crinkling (**b**) symptoms

The first disease symptom observed on RSMV-infected plants was mosaic stripes on leaves, which generally appeared at the tip of newly emerged leaves. In three repeated trials at 10 dpi, the incidence of mosaicism on the three cultivars was as follows: *indica* rice Meixiangzhan, 81.82, 43.75 and 5.56%; *japonica* rice Nipponbare, 56.52, 13.64 and 22.22%; and hybrid rice Wuyou 1179, 13.04, 30.00 and 3.85% (Fig. 2a). At 15 dpi, the incidence of mosaicism on the three tested varieties had increased significantly, to more than 80%. In the worst case, 90.91% of Meixiangzhan plants were affected. The mosaic symptoms of some infected plants later receded (Fig. 2a). As the disease developed, the proportion of mosaic leaves increased first and then decreased (Additional file 4: Figure S4). SPAD values, representing chlorophyll content, were always lower in infected plants than in healthy ones; the largest differences between infected and healthy plants were observed from 5 to 10 dpi and 30 to 45 dpi, and the smallest difference was from 15 to 25 dpi (Additional file 5: Figure S5).

Dwarfing was first observed at 15 dpi. By the end of the experiment, infected plants of the three tested varieties were approximately 80% of healthy control plants (Additional file 6: Figure S6). With the development of the disease, the dwarfing phenotype became increasingly obvious, especially in Wuyou 1179. At 25 dpi, the heights of infected plants were less than 80% of healthy control plants. Dwarfing was most pronounced in Meixiangzhan and Nipponbare during later disease stages. At 45 dpi, heights of Meixiangzhan and Nipponbare were respectively 88.04 and 79.53% of those of healthy control plants (Additional file 6: Figure S6).

Leaf crinkling was observed from 15 to 20 dpi in some infected plants. The emergence time of this symptom varied slightly among varieties, appearing first in Nipponbare, secondly in Meixiangzhan and finally in Wuyou 1179 (Figs. 2b and Additional file 7: Figure S7). At 20 dpi, the incidence of leaf crinkling based on three replicates was 34.78, 18.75 and 11.11% in Meixiangzhan, 13.04, 13.64 and 11.11% in Nipponbare, and 21.74%. 15.00 and 7.69% in Wuyou 1179 (Fig. 2b). At 45 dpi, the incidence of this symptom was significantly increased, reaching more than 60%, with that of Nipponbare and Wuyou 1179 at or above 90% (95.45 and 90.00%, respectively) (Fig. 2b). After an initial increase, the proportion of crinkled leaves per plant declined and then increased. As the disease developed, some leaf crinkling symptoms also receded (Additional file 8: Figure S8).

### Symptoms associated with rice yield and yield losses

**Fig. 3:**
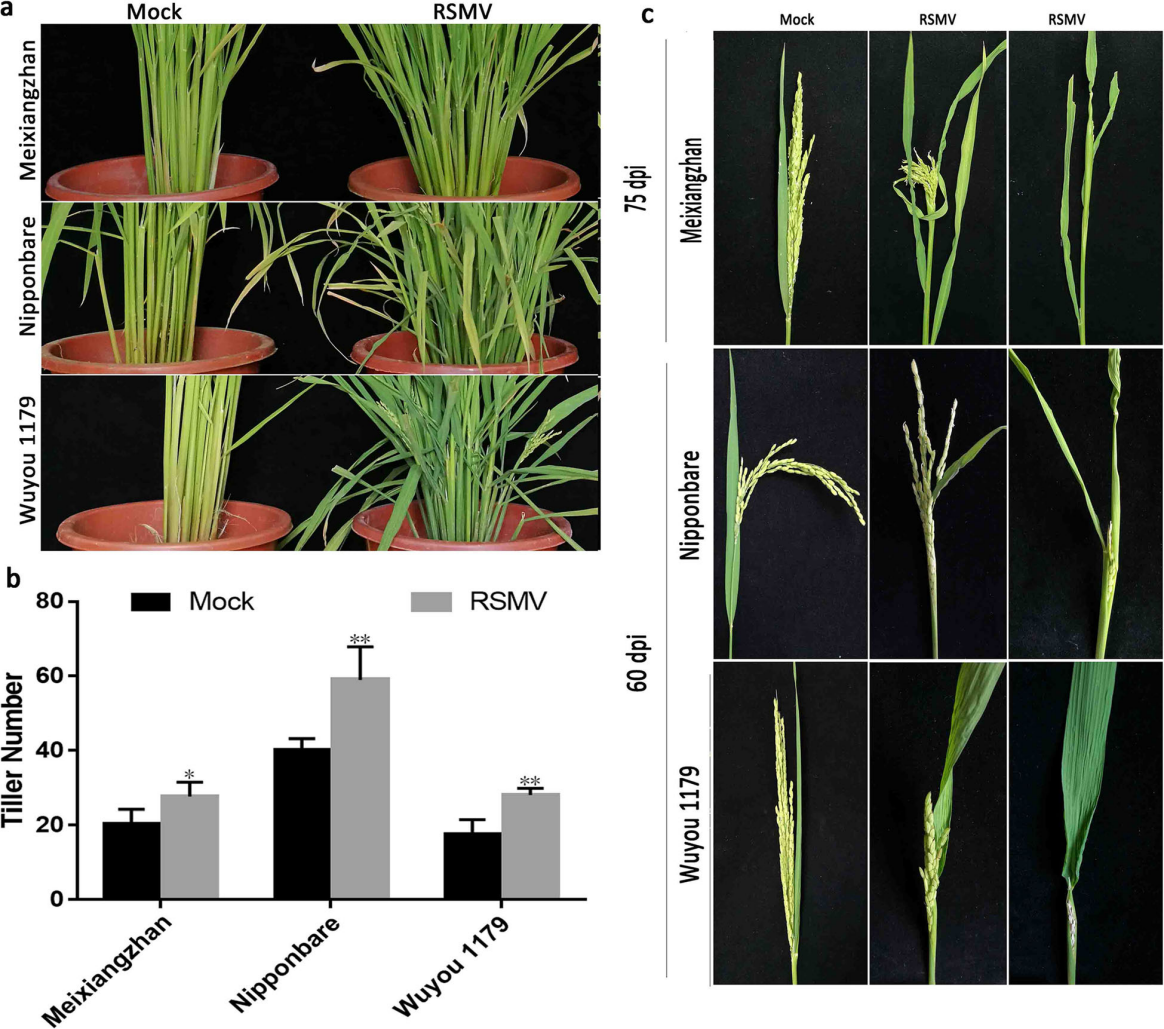
Symptoms related to yield loss in three RSMV-infected varieties. a–c Tiller appearance (**a**), tiller number (**b**) and extent of heading (**c**) of the three varieties at 60 dpi (Tiller numbers: Meixiangzhan, df = 28, SD = 1.31, *p* < 0.0001; Nipponbare, df = 17.278, SD = 2.23, *p* < 0.0001; Wuyou 1179, df = 20.087, SD = 1.04, *p* < 0.0001) Values in b are means ± s.d. (*n* = 15 plants). Significant differences are indicated by asterisks: *, significant (*P* < 0.05); **, highly significant (*P* < 0.01)

The number of tillers in the three RSMV-infected varieties increased significantly: by 40.00% in Meixiangzhan, 47.50% in Nipponbare and 55.56% in Wuyou 1179 (Figs. 3a–b and Additional file 9: Figure S9). Among the three varieties, these differences were not significant. In infected plants, heading time was significantly delayed, and heading was incomplete (Fig. 3c). When 100% of healthy control plants had undergone heading, the heading incidence of Meixiangzhan was 42.86% (Experiment II) and 72.73% (Experiment III); that of Nipponbare was only 84.38% (II) and 58.33% (III), while that of Wuyou 1179 was 34.78% (II) and 5.26% (III) (Table 1).

**Table 1:** Heading characteristics of three rice varieties after infection with RSMV

Varieties	Days post infection	Treatment	Number of heading plants	Number of all plants	The heading incidence (%)
Meixiangzhan	70dpi	CK	6	6	100.00
		RSMV	6	14	42.86
	75dpi	CK	22	22	100.00
		RSMV	16	22	72.73
Nipponbare	50dpi	CK	14	14	100.00
		RSMV	27	32	84.38
	55dpi	CK	10	10	100.00
		RSMV	14	24	58.33
Wuyou 1179	60dpi	CK	12	12	100.00
		RSMV	8	23	34.78
	70dpi	CK	6	6	100.00
		RSMV	1	19	5.26

**Fig. 4:**
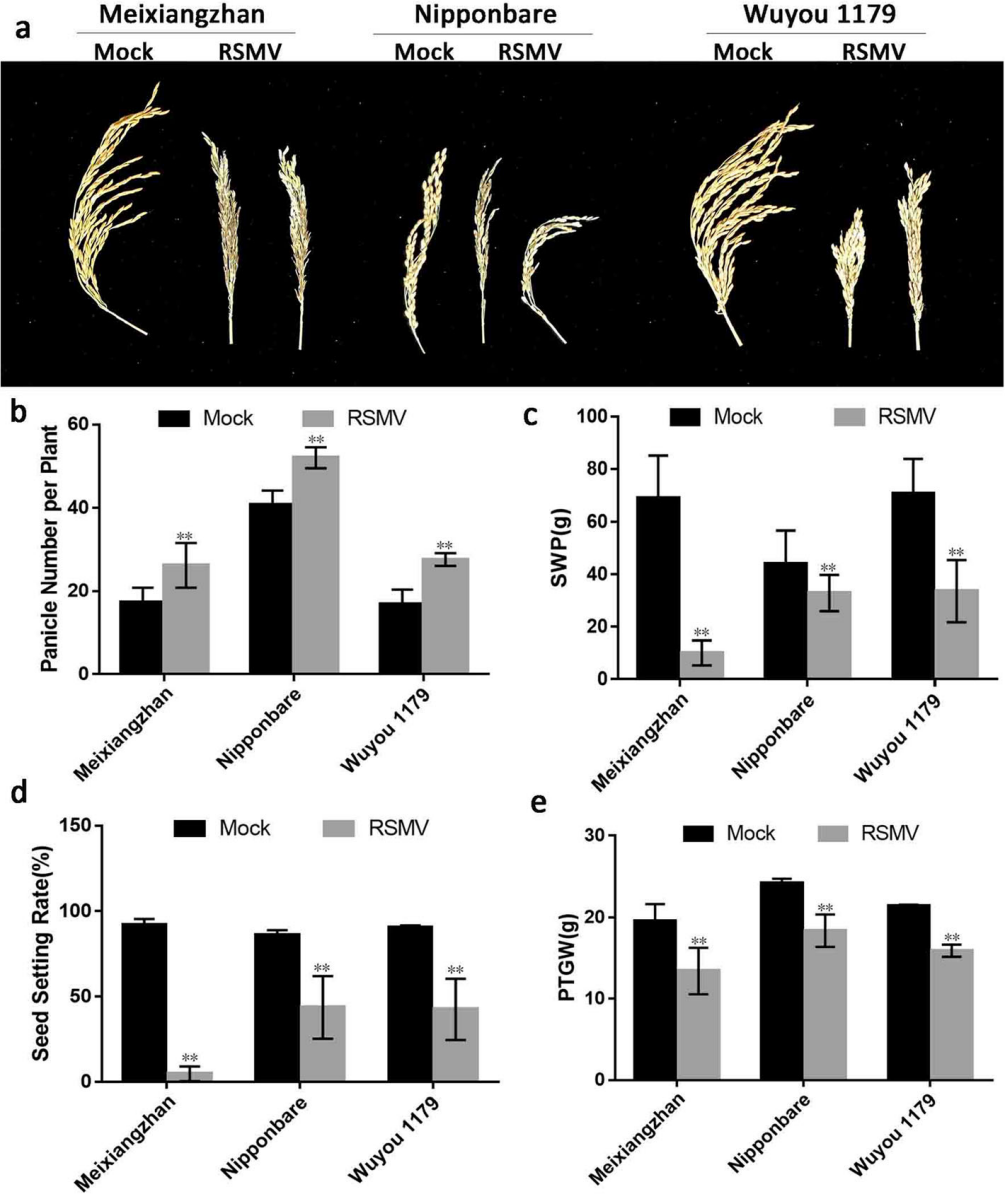
Yield measurements of three RSMV-infected rice varieties. **a** Ripened panicles. **b** Panicle number per plants (Meixiangzhan: df = 23.848, SD = 1.52, *p* < 0.0001; Nipponbare: df = 28, SD = 1.01, *p* < 0.0001; Wuyou 1179: df = 18.910, SD = 0.93, *p* < 0.0001). **c** Spike weight per plant (Meixiangzhan: df = 16.348, SD = 4.08, *p* < 0.0001; Nipponbare: df = 21.835, SD = 3.43, *p* = 0.003 < 0.01; Wuyou 1179: df = 28, SD = 4.26, *p* < 0.0001). **d** Seed setting rate (df = 1, *p* = 0.009 < 0.01). **e** 1000-kernel weight (Meixiangzhan: df = 28, SD = 0.84, *p* < 0.0001; Nipponbare: df = 28, SD = 0.49, *p* < 0.0001; Wuyou 1179: df = 15.270, SD = 0.18, *p* < 0.0001). Values in b–e are means ± s.d. (b–e, *n* = 15 plants). Significant differences are indicated by asterisks: *, significant (*P* < 0.05); **, highly significant (*P* < 0.01)

As the number of tillers increased, the number of rice panicles in RSMV-infected plants also increased. Average numbers of panicles per plant in the three tested varieties were 150.57% (Meixiangzhan), 127.45% (Nipponbare) and 162.29% (Wuyou 1179) of those of healthy plants (Figs. 4b and Additional file 10: Figure S10). In contrast to the increase in panicle numbers, panicle lengths of infected plants were significantly decreased, being only 65.47% (Meixiangzhan), 78.36% (Nipponbare) and 59.08% (Wuyou 1179) of those of healthy ones (Additional file 11: Figure S11). In addition, the panicles of Meixiangzhan and Wuyou 1179 were arranged in short clusters (Fig. 4a).

Single panicle weights of the three RSMV-infected varieties were 10.03 g (Maixiangzhan), 32.89 g (Nipponbare) and 33.58 g (Wuyou 1179), which were only 14.58, 74.47, and 47.58% of those of healthy controls. This difference was extremely significant (Fig. 4c). Among the three varieties, Meixiangzhan exhibited the most drastic reduction, up to 85.42%, while the reductions in Wuyou 1179 and Nipponbare were 52.43 and 25.53%, respectively. Among Meixiangzhan and Nipponbare, the difference was significant (Additional file 12: Figure S12). Compared with the control, seed setting rates of the diseased rice plantlets were also significantly reduced, by 94.86% (Maixiangzhan), 49.32% (Nipponbare) and 53.06% (Wuyou 1179). The differences among the varieties were significant (Figs. 4d and Additional file 13: Figure S13).

The 1000-kernel weights of RSMV-infected plants were significantly reduced: to 68.44% (Maixiangzhan), 76.14% (Nipponbare) and 74.35% (Wuyou 1179) of those of healthy plants. The level of reduction differed significantly among varieties (Figs. 4e and Additional file 14: Figure S14).

## Discussion

The three rice varieties infected with RSMV exhibited slight dwarfing, striped mosaicism, stiff, crinkled and even twisted leaves, an increased number of tillers, delayed heading, cluster-shaped shortening of panicles and mostly unfilled grains. Among these symptoms, mosaicism appeared earlier and crinkling later, with both symptoms receding in some infected plants. Yield losses due to RSMV also differed among varieties, with the most serious yield reduction observed in *indica* cultivar Meixiangzhan, followed by hybrid Wuyou 1179 and then *japonica* cultivar Nipponbare. Compared with healthy plants, infected varieties had single panicle weights, seed setting rates and 1000-kernel weights that were reduced by 85.42, 94.85 and 31.56% (Meixiangzhan); 52.43, 53.06 and 25.65% (Wuyou 1179); and 25.53, 49.32 and 23.86% (Nipponbare), respectively.

RSMV is a recently identified, tentative new rice virus. Because published studies on the virus are limited, the symptoms of RSMV are still relatively unknown. In the present investigation, we studied the symptoms of RSMV-infected rice by simulating field environmental conditions, with the goal of providing a biological basis for a follow-up theoretical study of RSMV. According to our research, the symptoms of RSMV can be clearly recognized in the field, thus providing a reference for early identification of the disease and timely prevention and control measures according to the disease situation.

Although the leaf stripe mosaic symptom of RSMV-infected plants is similar to that of rice infected with rice stripe virus (RSV, transmitted by the small brown planthopper (SBPH; *Laodelphax striatellus*), *Terthron albovittatum*, *Unkanodes sapporonus* and *Unkanodes albifascia*), some characteristic differences exist. At the early infection phase, both viruses cause mottled stripes on leaves of infected rice; at the later infection stage, however, significant differences are observed. In particular, the mosaic symptoms of RSMV gradually diminish or even disappear, whereas mottled leaves infected with RSV whiten or even wither [9]. In addition, RSV-infected leaves exhibit a drooping phenotype, while RSMV-infected rice has erect leaves that are more rigid than those of healthy plants. This latter characteristic is similar to the symptoms of rice dwarf virus (RDV, transmitted in a persistent manner by *Nephotettix cincticeps*, *N. nigropictus*, *Recilia dorsalis*, and some other *Nephotettix* spp.), rice gall dwarf virus (RGDV, transmitted in a persistent manner by *N. cincticeps*, *N. nigropictus*, *Recilia dorsalis*, and some other *Nephotettix* spp.), rice black-streaked dwarf virus (RBSDV, transmitted in a persistent manner primarily by the planthopper, *Laodelphax striatellus*, *U. sapporonus* and *Ribautodelphax albifascia*) and southern rice black-streaked dwarf virus (SRBSDV, transmitted by *Sogatella furcifera* in a persistent propagative manner) [3, 5, 6, 10]. Leaf tensile strength and stiffness, which mainly depend on cellulose, hemicellulose and lignin contents, are also related to hormonal changes in plants [11–13]. In our study, cellulose, hemicellulose and lignin contents of leaves of RSMV-infected plants changed significantly. The increased tiller number symptom of RSMV is comparable to that of rice bunchy stunt virus (RBSV, transmitted in a persistent manner by *Nephotettix cincticeps* and *N. virescens*), RDV and rice grass stunt virus (RGSV, transmitted in a persistent manner by the brown planthopper *Nilaparvata lugens* and by two other *Nilaparvata* spp.) and differs from that of RGDV and rice yellow stunt virus (RYSV, transmitted in a persistent manner by *Nephotettix cincticeps*, *N. nigropictus*, and *N. virescens*) [3, 14–18]. Similar to the effects of many rice virus diseases, RSMV-infected plants exhibit dwarfing; however, the degree of dwarfing induced by RSMV is far less than that from SRBSDV, RGDV, RBSDV, RDV, rice ragged stunt virus (RRSV, transmitted in a persistent manner by the brown planthopper, *Nilaparvata lugens*) and RYSV but is similar to that caused by RSV [6, 19–23].

The mosaicism and crinkling symptoms observed in RSMV-infected leaves may recede, which is similar to the progression of leaf yellowing caused by RYSV [24, 25]. Both viruses are species in the *Rhabdoviridae* family, the former in the genus *Cytorhabdovirus* and the latter in *Nucleorhabdovirus.* More interestingly, leaf symptoms caused by barely yellow striate mosaic virus (BYSMV; genus *Cytorhabdovirus*, family *Rhabdoviridae*) also recede [26, 27]. Whether recession is a common feature of *Rhabdoviridae*-caused diseases thus deserves further study [28–30].

Rice virus diseases have the characteristics of burstiness and destructiveness, which cause intermittent disasters in eastern and southeastern Asia. For example, RSV and RBSDV transmitted by the small brown planthopper *Laodelphax striatellus* caused serious damage to rice production in the 1960s and 2000s [3, 10, 31–35], and RDV and RYSV transmitted by leafhoppers in the 1980s are widespread in Vietnam, China and Japan [3]. Since 2009, SRBSDV has rapidly spread and has caused serious rice losses in northern Vietnam and southern China [36]. RSMV is transmitted by the leafhopper *R. dorsalis* in a persistent-propagative manner [5]. Since its discovery in 2015, the virus has spread rapidly in southern China [35]. More intense disease monitoring is thus needed. The progression of disease symptoms revealed in our study provide useful information for disease field investigations.

## Conclusions

RSMV-infected plants exhibited slight dwarfing, striped mosaicism, stiff, crinkled or even twisted leaves, an increased number of tillers, delayed heading, cluster-shaped shortening of panicles and mostly unfilled grains. Among these symptoms, mosaicism appeared earlier and crinkling later, with both symptoms later receding in some infected plants. Yield losses due to RSMV also differed among varieties. *Indica* rice (cv. Meixiangzhan) experienced the most drastic yield reduction, followed by hybrid *indica* rice (cv. Wuyou 1179) and then *japonica* (cv. Nipponbare). The results of this study provide basic data for field investigations, the formulation of prevention and control strategies and further study of the pathogenesis of RSMV.

## Supplementary information

**Additional file 1: Figure S1.** Degree of dwarfing in three RSMV-infected rice varieties.

**Additional file 2: Figure S2.** Percentage decrease in SPAD values of three RSMV-infected rice varieties.

**Additional file 3: Figure S3.** Glucose contents of three RSMV-infected rice varieties.

**Additional file 4: Figure S4.** Rates of leaf mosaicism of three RSMV-infected rice varieties.

**Additional file 5: Figure S5.** SPAD values of three RSMV-infected rice varieties.

**Additional file 6: Figure S6.** Plant heights of three RSMV-infected rice varieties.

**Additional file 7: Figure S7.** Leaves of three RSMV-infected rice varieties exhibiting crinkled and twisted phenotypes at different infection stages.

**Additional file 8: Figure S8.** Percentages of crinkled leaves of three RSMV-infected rice varieties.

**Additional file 9: Figure S9.** Percentage increase in tiller number of three RSMV-infected rice varieties. Error bars represent standard deviations.

**Additional file 10: Figure S10.** Percentage increase in the effective panicle number of three RSMV-infected rice varieties.

**Additional file 11: Figure S11.** Percentage reduction in panicle lengths of three RSMV-infected rice varieties.

**Additional file 12: Figure S12.** Percentage decrease in the single panicle weight per plant of three RSMV-infected rice varieties.

**Additional file 13: Figure S13.** Percentage decrease in seed setting rates of three RSMV-infected rice varieties.

**Additional file 14: Figure S14.** Percentage decrease in the 1000-kernel weight of three RSMV-infected rice varieties.

**Additional file 15: Table S1.** Contents (%) of compounds related to leaf stiffness in three RSMV-infected rice varieties.

## Data Availability

Not applicable.

## Abbreviations

BYSMV: barely yellow striate mosaic virus
dpi: day post inoculation.
RBSDV: Rice black-streaked dwarf virus
RBSV: Rice bunchy stunt virus
RDV: Rice dwarf virus
RGDV: Rice gall dwarf virus
RGSV: Rice grass stunt virus
RRSV: Rice ragged stunt virus
RRSV: Rice ragged stunt virus
RSMV: Rice stripe mosaic virus
RSV: Rice stripe virus
RT-PCR: Reverse transcription polymerase chain reaction
RYSV: Rice yellow stunt virus
SRBSDV: Southern rice black-streaked dwarf virus
