# Supplementary figures and images for: Symptoms and yield loss caused by rice stripe mosaic virus
Source: Virol J. 2019 Nov 27;16:145. doi: 10.1186/s12985-019-1240-7 (PMC6880357; doi:10.1186/s12985-019-1240-7)

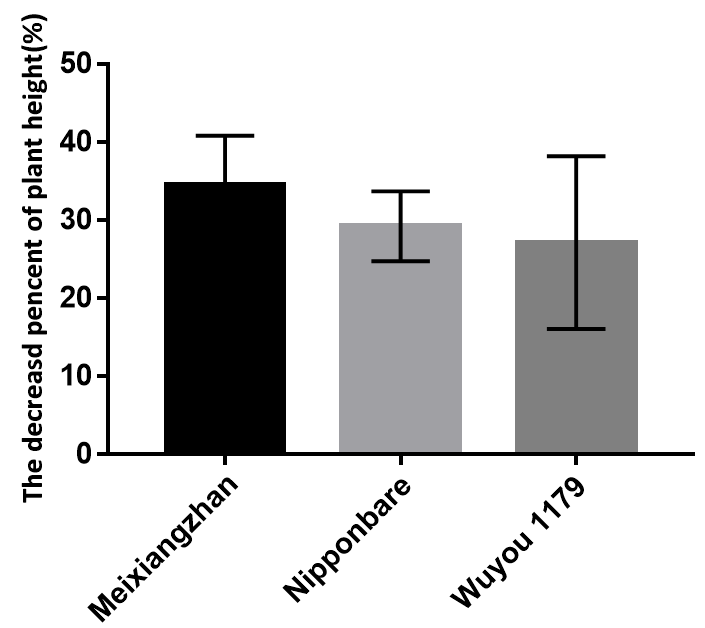

Supplement: Supplementary file 1 — Additional file 1: Figure S1. Degree of dwarfing in three RSMV-infected rice varieties. [file 12985_2019_1240_MOESM1_ESM.png]

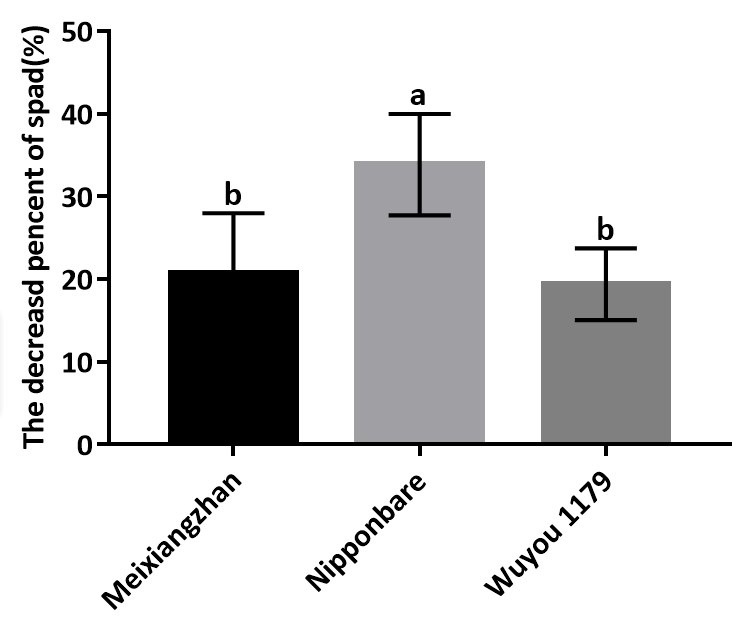

Supplement: Supplementary file 2 — Additional file 2: Figure S2. Percentage decrease in SPAD values of three RSMV-infected rice varieties. [file 12985_2019_1240_MOESM2_ESM.png]

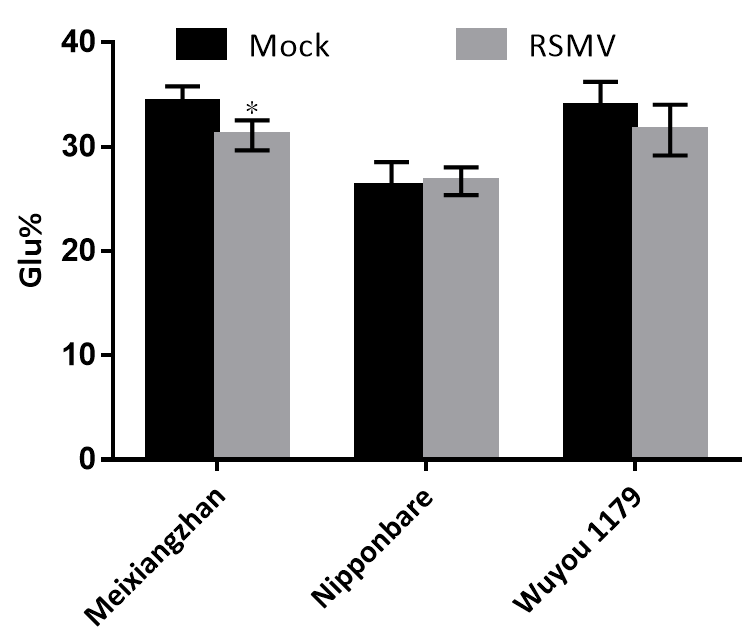

Supplement: Supplementary file 3 — Additional file 3: Figure S3. Glucose contents of three RSMV-infected rice varieties. [file 12985_2019_1240_MOESM3_ESM.png]

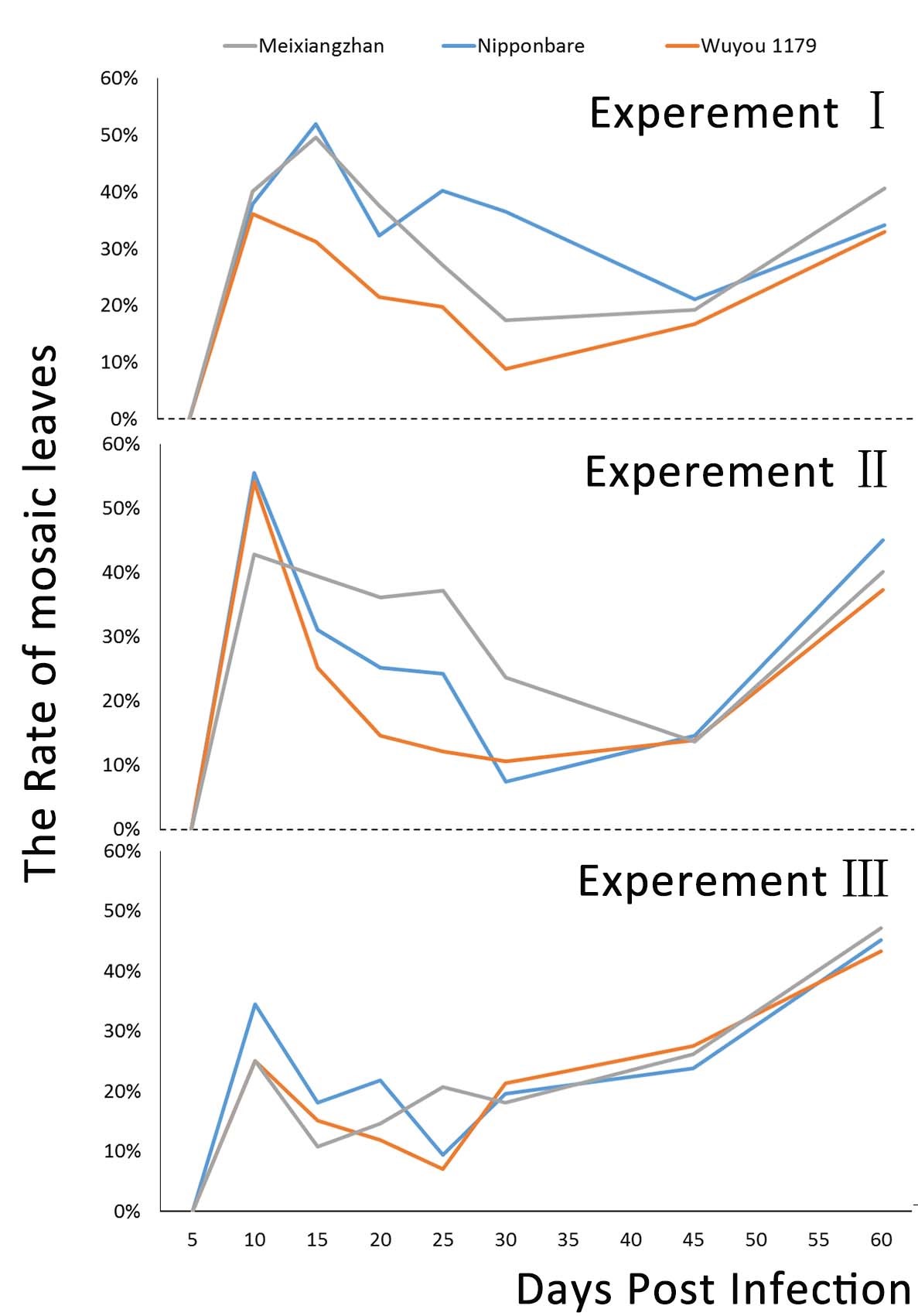

Supplement: Supplementary file 4 — Additional file 4: Figure S4. Rates of leaf mosaicism of three RSMV-infected rice varieties. [file 12985_2019_1240_MOESM4_ESM.jpg]

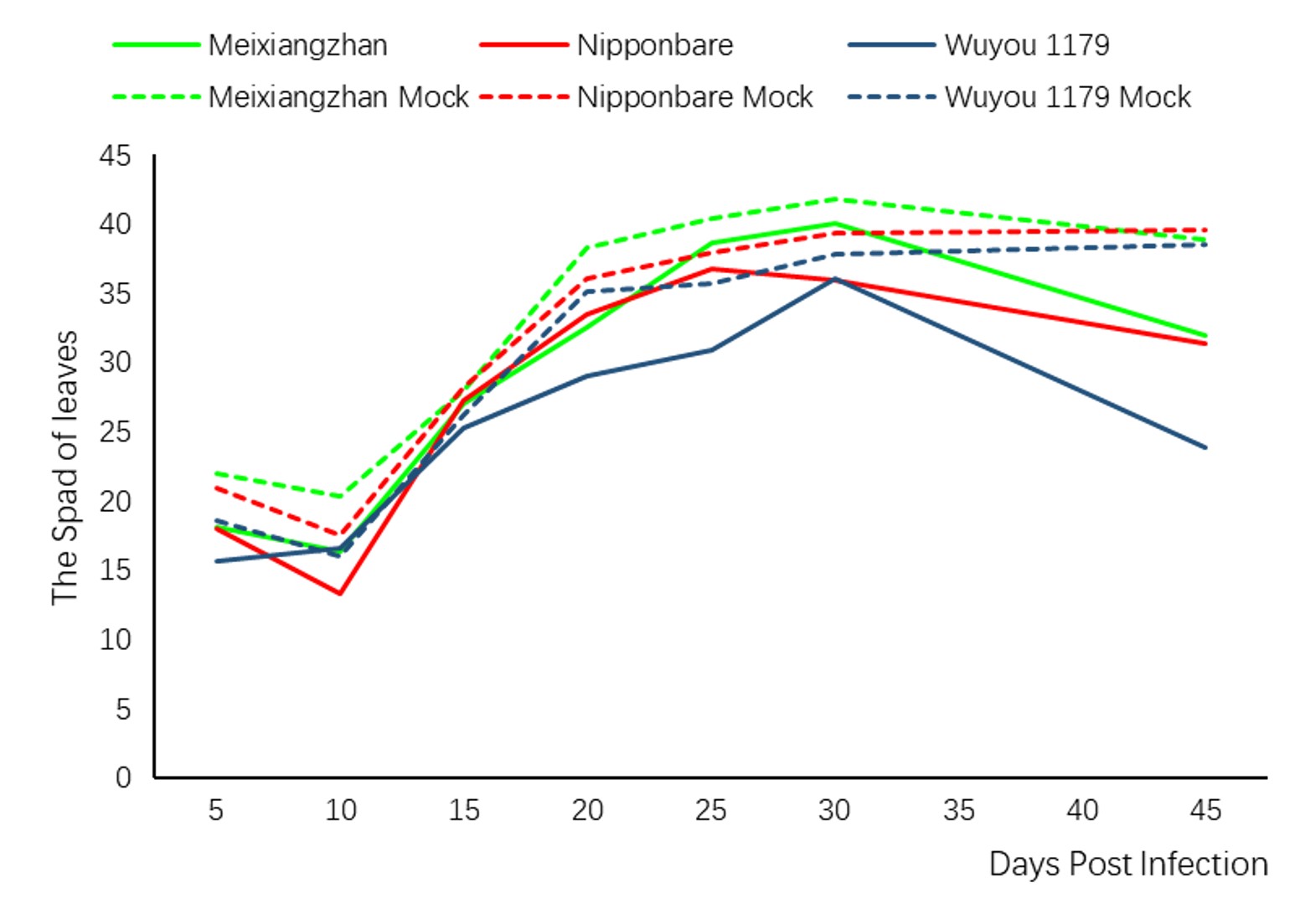

Supplement: Supplementary file 5 — Additional file 5: Figure S5. SPAD values of three RSMV-infected rice varieties. [file 12985_2019_1240_MOESM5_ESM.jpg]

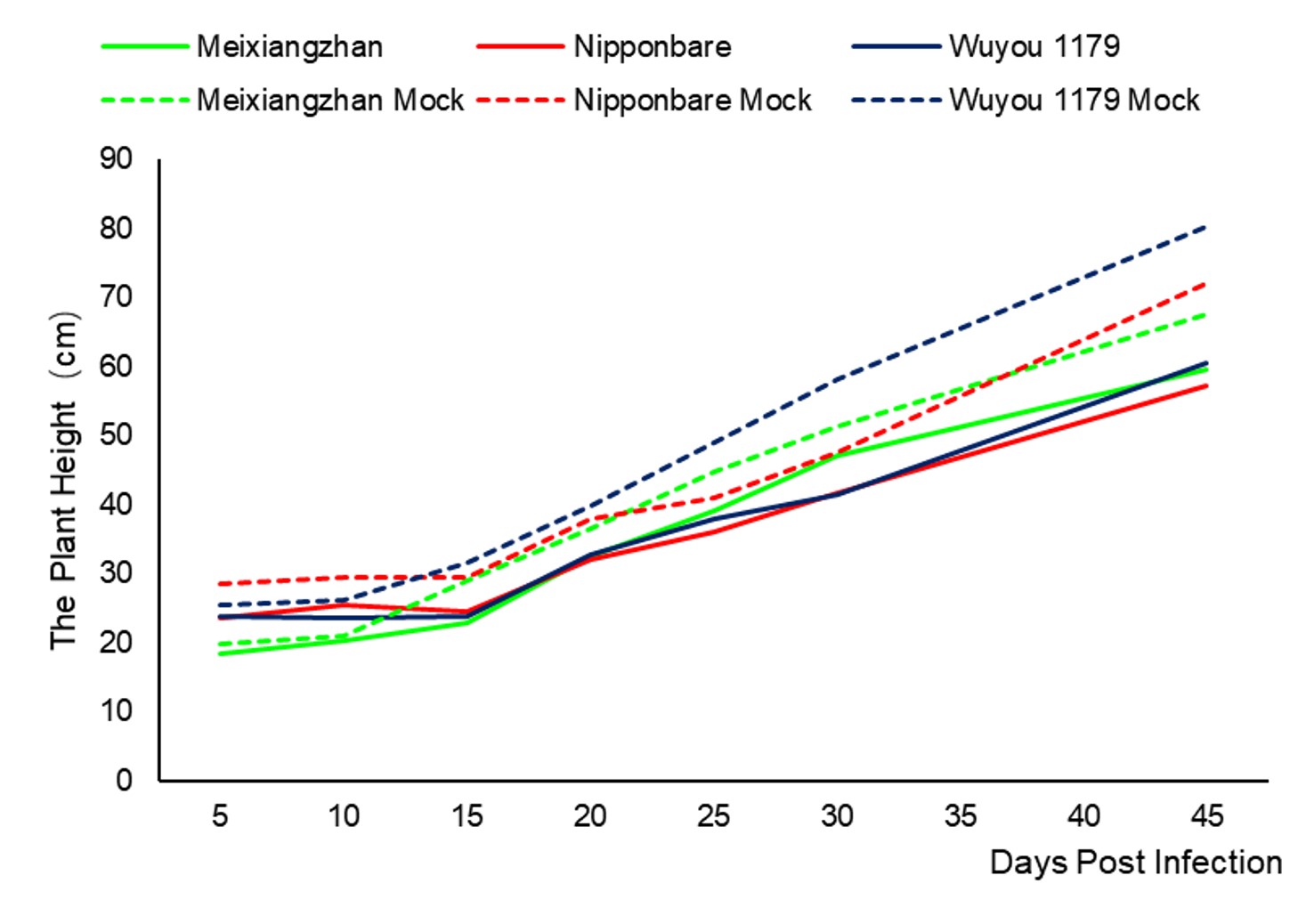

Supplement: Supplementary file 6 — Additional file 6: Figure S6. Plant heights of three RSMV-infected rice varieties. [file 12985_2019_1240_MOESM6_ESM.jpg]

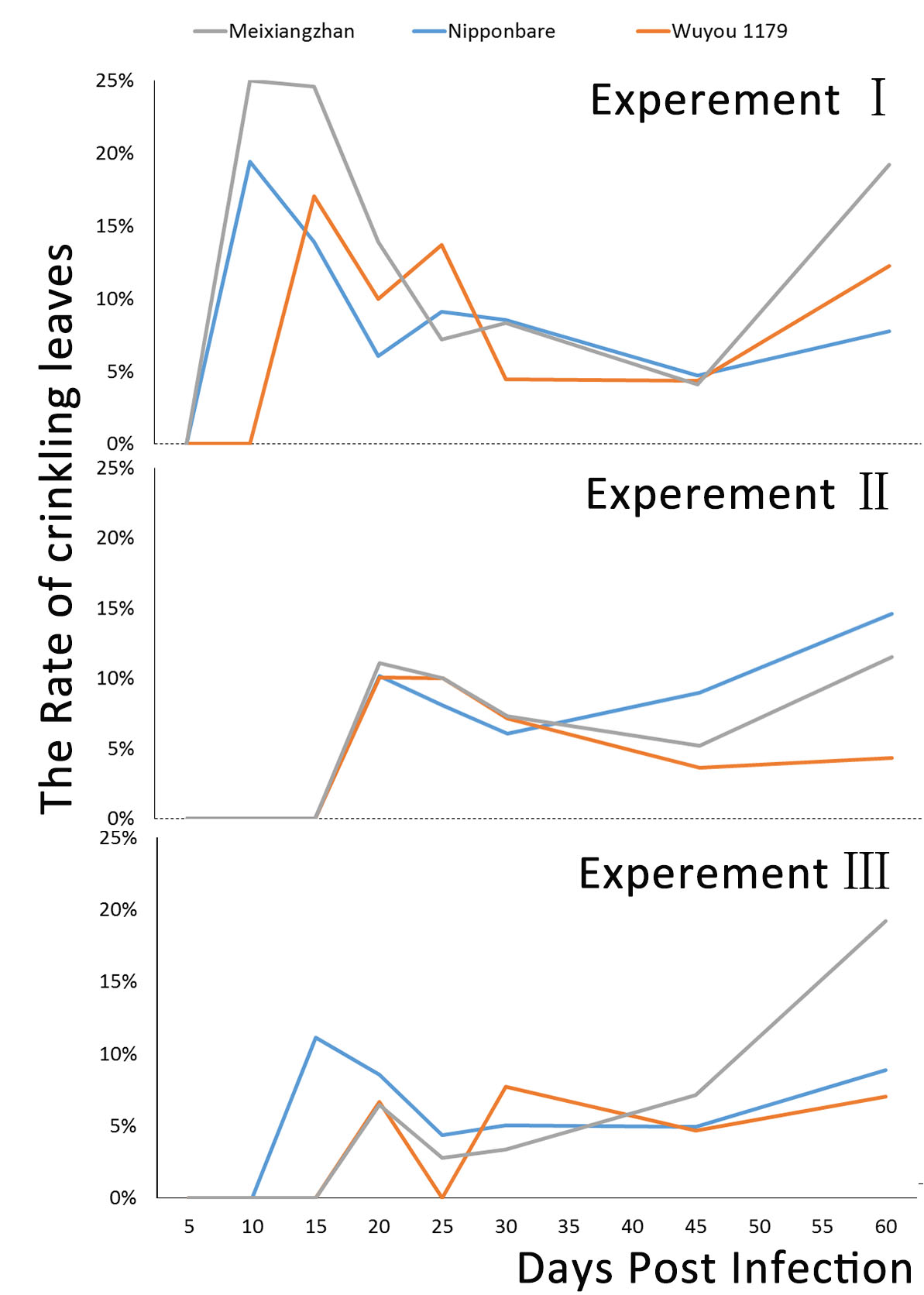

Supplement: Supplementary file 8 — Additional file 8: Figure S8. Percentages of crinkled leaves of three RSMV-infected rice varieties. [file 12985_2019_1240_MOESM8_ESM.jpg]

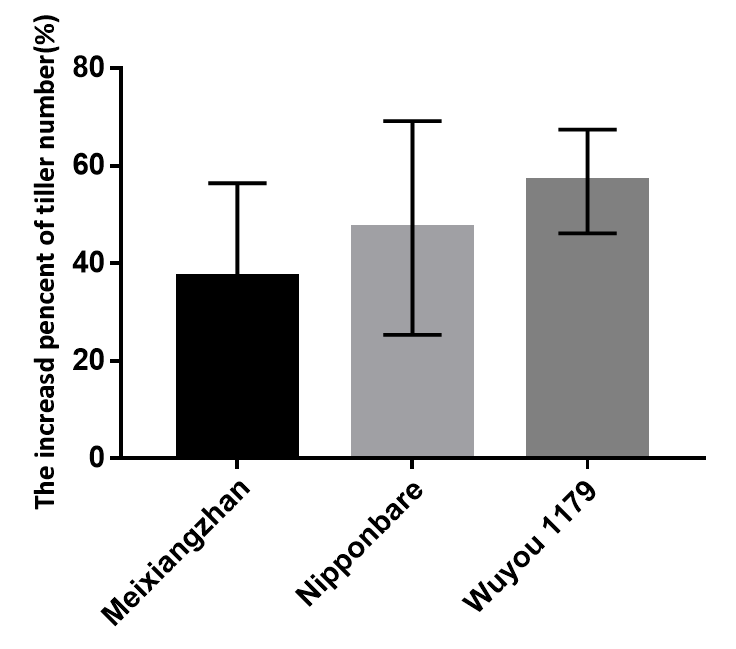

Supplement: Supplementary file 9 — Additional file 9: Figure S9. Percentage increase in tiller number of three RSMV-infected rice varieties. Error bars represent standard deviations. [file 12985_2019_1240_MOESM9_ESM.png]

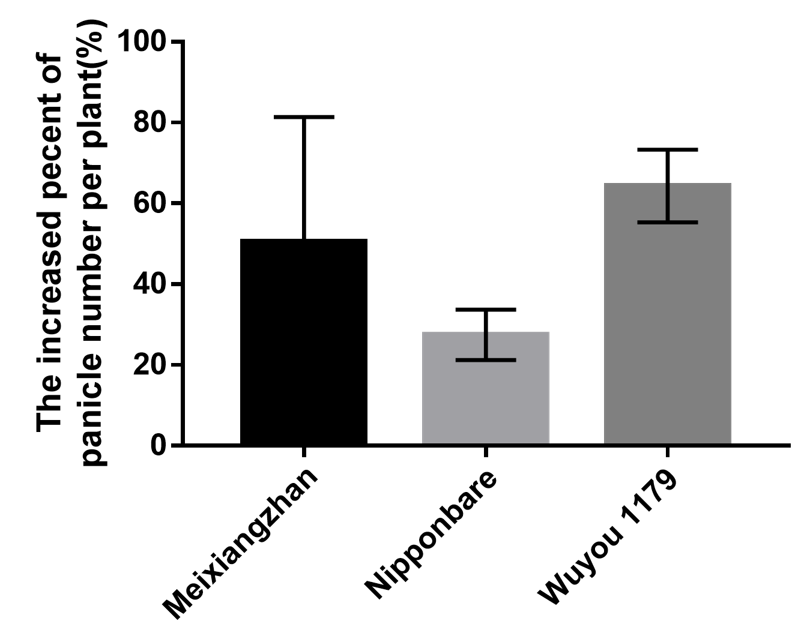

Supplement: Supplementary file 10 — Additional file 10: Figure S10. Percentage increase in the effective panicle number of three RSMV-infected rice varieties. [file 12985_2019_1240_MOESM10_ESM.png]

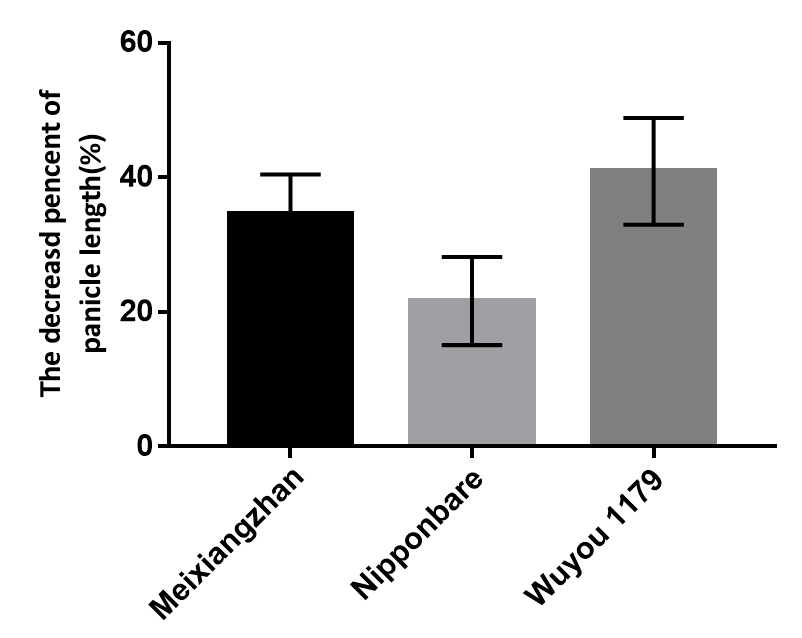

Supplement: Supplementary file 11 — Additional file 11: Figure S11. Percentage reduction in panicle lengths of three RSMV-infected rice varieties. [file 12985_2019_1240_MOESM11_ESM.png]

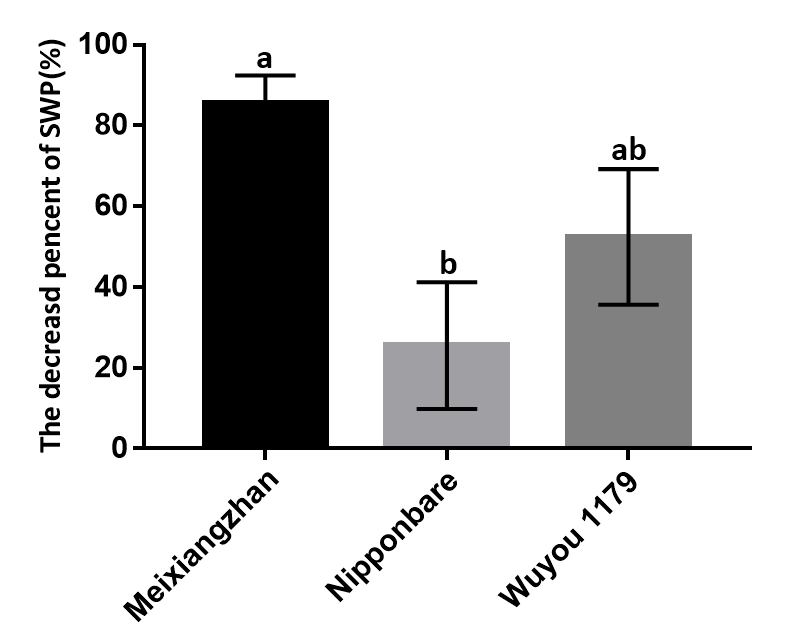

Supplement: Supplementary file 12 — Additional file 12: Figure S12. Percentage decrease in the single panicle weight per plant of three RSMV-infected rice varieties. [file 12985_2019_1240_MOESM12_ESM.png]

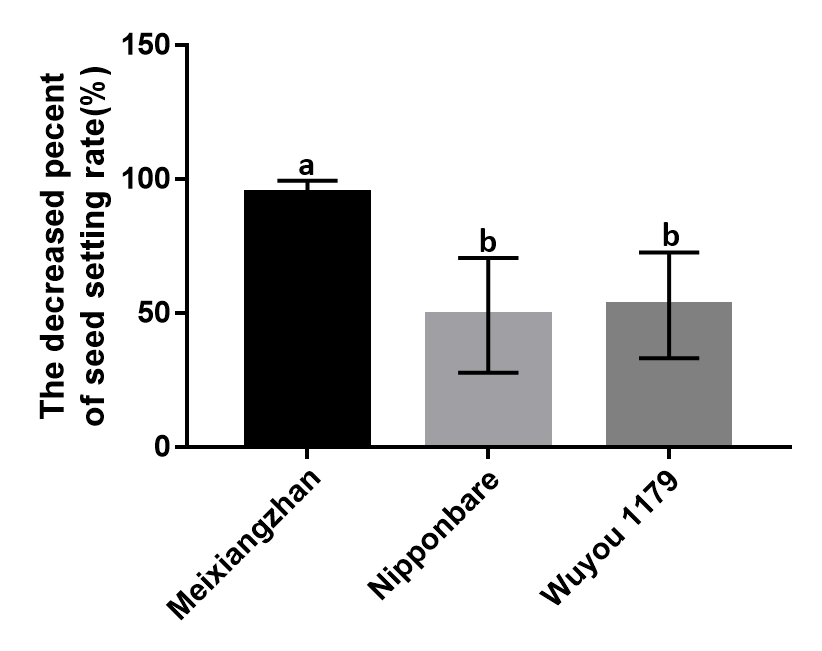

Supplement: Supplementary file 13 — Additional file 13: Figure S13. Percentage decrease in seed setting rates of three RSMV-infected rice varieties. [file 12985_2019_1240_MOESM13_ESM.png]

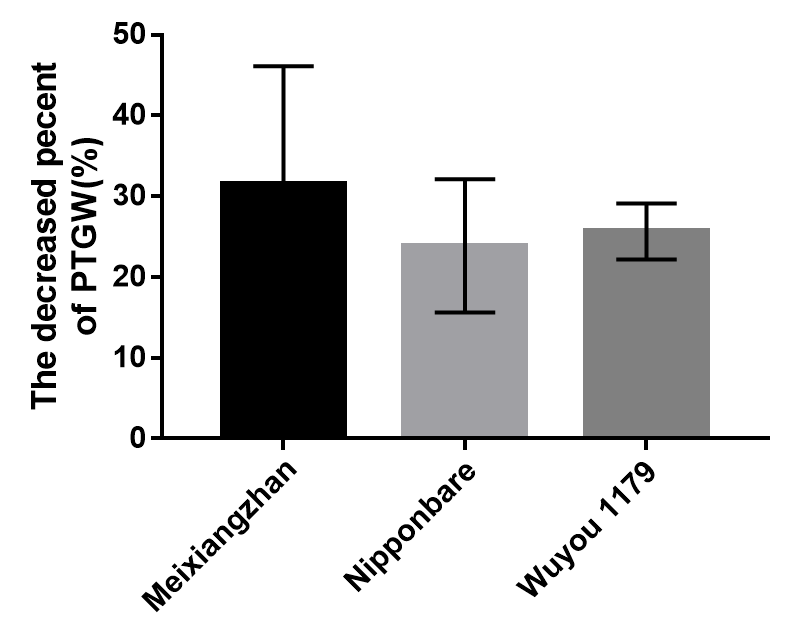

Supplement: Supplementary file 14 — Additional file 14: Figure S14. Percentage decrease in the 1000-kernel weight of three RSMV-infected rice varieties. [file 12985_2019_1240_MOESM14_ESM.png]
