# Supplementary material for: Symptoms and yield loss caused by rice stripe mosaic virus
Source: Virol J. 2019 Nov 27;16:145. doi: 10.1186/s12985-019-1240-7 (PMC6880357; doi:10.1186/s12985-019-1240-7)
Supplement: Supplementary file 15 — Additional file 15: Table S1. Contents (%) of compounds related to leaf stiffness in three RSMV-infected rice varieties. [file 12985_2019_1240_MOESM15_ESM.docx]

Table S1 Contents (%) of compounds related to leaf stiffness in three RSMV-infected rice varieties

| Traits | Meixiangzhan | |  | Nipponbare | |  | Wuyou 1179 | |
| --- | --- | --- | --- | --- | --- | --- | --- | --- |
|  | CK | RSMV |  | CK | RSMV |  | CK | RSMV |
| Glucose content (Glu%) | 34.31±1.22 | 31.11±1.16* |  | 26.27±1.87 | 26.72±1.08 |  | 33.94±1.89 | 31.61±2.00 |
| Xylose content (Xyl%) | 13.93±0.58 | 12.41±0.54* |  | 10.45±0.57 | 7.87±2.23* |  | 13.59±0.79 | 11.69±0.98 |
| Arabinose content (Ara%) | 7.47±0.93 | 7.41±0.76 |  | 5.46±0.16 | 6.19±0.46 |  | 6.61±0.35 | 6.85±0.66 |
| Galactose content (Gal%) | 1.57±0.03 | 1.66±0.01* |  | 1.63±0.06 | 1.63±0.09 |  | 1.58±0.02 | 1.66±0.02* |
| Cellulose content (%) | 37.74±1.34 | 34.23±1.28* |  | 28.90±2.06 | 29.39±1.19 |  | 37.34±2.08 | 34.77±2.19 |
| Hemicellulose content (%) | 22.97±1.31 | 21.48±1.19 |  | 17.54±0.75 | 15.69±1.97 |  | 21.78±1.12 | 20.21±1.64 |
| Acid-insoluble lignin content (AIL%) | 16.18±0.67 | 18.72±2.05* |  | 30.18±0.00 | 32.47±3.99 |  | 15.15±0.33 | 17.46±0.33* |
| Acid-soluble lignin (ASL%) | 1.49±0.08 | 1.49±0.06 |  | 1.13±0.06 | 1.19±0.03 |  | 1.45±0.08 | 1.51±0.05 |
| Lignin Content (%) | 17.67±0.64 | 20.21±2.02* |  | 31.39±0.05 | 33.67±3.97 |  | 16.6±0.25 | 18.97±0.37* |
